# Optimizing Outcomes in Flexible Ureteroscopy: A Narrative Review of Suction Techniques

**DOI:** 10.3390/jcm12082815

**Published:** 2023-04-11

**Authors:** Catalina Solano, Marie Chicaud, Stessy Kutchukian, Luigi Candela, Mariela Corrales, Frédéric Panthier, Steeve Doizi, Olivier Traxer

**Affiliations:** 1 GRC n 20, Groupe de Recherche Clinique sur la Lithiasis Urinaire, Hôpital Tenon, Sorbonne Université, 75020 Paris, France; catasolano84@gmail.com (C.S.); candela.luigi@hsr.it (L.C.); mariela_corrales_a@hotmail.com (M.C.); fredericpanthier@gmail.com (F.P.); steeve.doizi@gmail.com (S.D.); 2 Department of Endourology, Uroclin S.A.S., Medellín 50011, Colombia; 3 Department of Urology, Limoges University Hospital, 2 Avenue Martin Luther King, 87000 Limoges, France; marie.chicaud@hotmail.fr; 4 Department of Urology, Poitiers University Hospital, 2 Rue de la Milétrie, 86000 Poitiers, France; stessy.kutchukian@gmail.com; 5 Unit of Urology, Division of Experimental Oncology, Urological Research Institute (URI), IRCCS Ospedale San Raffaele, 20100 Milan, Italy

**Keywords:** suction, renal pressure, stone-free rate, retrograde intrarenal surgery, RIRS, stone disease

## Abstract

Objective: The aim of this review is to summarize the existing suction systems in flexible ureteroscopy (fURS) and to evaluate their effectiveness and safety. Methods: A narrative review was performed using the Pubmed and Web of Science Core Collection (WoSCC) databases. Additionally, we conducted a search on the Twitter platform. Studies including suctions systems in fURS were included. Editorials, letters and studies reporting intervention with semirigid ureteroscopy, PCNL and mPCNL were excluded. Results: A total of 12 studies were included in this review. These studies comprised one in vitro study, one ex vivo study, one experimental study and eight cohort studies. The Pubmed and WoSCC searches identified three suction techniques (Irrigation/Suctioning system with control of pressure, suction ureteral access sheath (sUAS) and direct in scope suction (DISS)), and the Twitter search identified four of them. The overall results showed that suction is an effective and safe technique that improves stone-free rates, reduces operative time and limits complication rates after fURS. Conclusions: The use of suctioning during common endourological procedures has been shown to improve safety and efficacy in several indications. However, randomized controlled trials are needed to confirm this.

## 1. Introduction

Urolithiasis is a common disorder with high and increasing prevalence in industrialized countries [1]. Among the treatments, we found shock wave lithotripsy (SWL), retrograde intrarenal surgery (RIRS) and percutaneous nephrolithotomy (PCNL). According to current guidelines, RIRS is recommended as the first or second choice for the treatment of kidney stones, regardless of the size of the stone; this includes stones larger than 2 cm [2]. Despite the benefits of using newer generation flexible ureterorenoscopes, Holmium: YAG laser lithotripsy (Ho:YAG) and Thulium fiber laser (TFL) for stone removal, it can be difficult to achieve a high success rate in removing >2 cm stones. Studies have reported stone-free rates (SFRs) ranging from 56.7% in a single procedure to 89.3% after 1.6 procedures [3].

These procedures aim to achieve a high SFR, but the presence of residual fragments (RFs) can be a problem and may lead to the need for additional interventions. The concept of clinically insignificant residual fragments (CIRFs) has been proposed, but there is currently no gold standard for managing, clearing or preventing RFs [4].

For over 25 years, suction has been utilized in endourology, often in conjunction with ultrasound and ballistic devices, to facilitate the removal of renal stones during PCNL [5]. One advantage of using a suction device during a RIRS procedure is that it can help to lower the temperature and pressure inside the kidneys, potentially reducing the risk of infection and improving surgical outcomes [6]. The specific techniques used for fragmenting or dusting stones during flexible ureteroscopy (fURS), such as a stone vacuum or steerable multi-lumen irrigation/aspiration device, vary according to the specific circumstances, such as the type of laser being used, and the surgeon’s preferences.

In recent years, the use of social media (SoMe) in the field of urology has grown significantly; urologists crowdsource for clinical cases and obtain input from colleagues, and trainee urologists are also using SoMe as a tool for surgical education [7]. Given that, we decided to report Twitter^®^ findings relevant to the topic of our review. The aim of this review is to summarize recent developments in suction systems for fURS and to evaluate their effectiveness in improving surgical outcomes and reducing complications.

## 2. Materials and Methods

We did a narrative review using the PubMed database and Web of Science Core Collection (WosCC) with no time period restriction from inception until December 2022. Data were reviewed independently by one author (C.S).

The keywords used were the following: “aspiration”, “suction”, “suctioning”, “endourology”, “ureteroscopy”, “urolithiasis” and “RIRS”. Boolean operators [AND, OR] were used to refine the search. The references of each included study were also reviewed, and no language restrictions were applied. Only studies focusing on suction devices during fURS were included. Editorials, letters, and studies reporting intervention with semirigid ureteroscopy, PCNL and mPCNL were excluded.

We also conducted a search on the Twitter^®^ platform (http://www.twitter.com) by two authors (CS and K) in the same time period, with the following keywords: “suction”, “scope suction”, “suction device”, “aspiration”, “RIRS suction”, “Ureteral suction”. For this study, all findings were carefully reviewed by two authors (CS and K). The tweets included contain important information related to the topic being studied.

Data extraction: The data were extracted using an Excel spreadsheet: author, year, center, study design, sample, groups of comparisons, suction modality, operative time, stone free rate, definition of stone free rate and complications.

Methodological quality and risk bias: The following is a narrative review without assessment of the quality of the articles.

Ethics statement, research involving Human Participants and/or Animals and Informed consent: The study was exempt from requiring the participants’ written informed consent because this is a narrative review.

## 3. Results

### 3.1. Literature Search

Our literature search identified 253 articles of interest, of which 132 abstracts and manuscripts were reviewed after duplication exclusion. This process identified that only 12 studies mentioned fURS and suction or topics related to it, and those studies eligible for the present narrative review comprised 4 laboratory studies (1 in vitro, 2 ex vivo and 1 preclinical) and 8 clinical studies. To better meet the main purpose of the present report, the laboratory and clinical results obtained were divided into different sections according to their main purpose (Table 1 and Table 2).

In vitro and experimental studies [8,9,10,11].

All in vitro and experimental results are summarized in Table 1.

In in vitro studies, Schneider et al. used a 10 mL Luer Lock syringe that was connected to a fiber optic ureteroscope (Karl Storz Flex X2, Storz, Germany) with a 3.6 working channel. They observed that the mean percentage of stone fragments suctioned through the ureteroscope was 86% (*p* = 0.973) for both groups (<0.5 and <1.0 mm); however, during suctioning, stones in the <1 mm group were trapped 4 more time compared with <0.5% (*p* = 0.02) [8]. Suctioning often resulted in the trapping of fragments, which could be time-consuming to remove.

Jiang et al. conducted an experimental study in 72 ex vivo porcine kidney models, divided into 12 groups of 6. The groups were defined by the laser type (Ho:YAG, Holmium with Moses technology and Thulium laser), the presence or absence of suction during the procedure and the presence or absence of a 14F ureteral access sheath [UAS]. Stones were treated at 16W using dusting settings for up to 20 min. Ho:YAG: 0.4 J–40 Hz, Ho:YAG-MOSES: 0.2 J–80 Hz and sTFL:0.2 J–80 Hz. The combination of TFL, 14 Fr UAS and aspiration resulted in 94% of stone fragments being cleared, with the remaining 6% being less than 2 mm in size compared with the other techniques [9].

Chen et al. conducted an experimental study in 20 porcine kidneys. They used a flexible vacuum assistant UAS (FV-UAS) 12/14F–46 cm, which perceives the change in intrarenal pressure (IRP), compared with traditional UAS groups at different irrigation fluid velocities of 30, 50, 80 and 100 mL/min. The FV-UAS (12/14F + RIRS + vacuum suction device) reported the mean stone volume clearance rates were 98.5% vs. traditional UAS groups, which were 95.9% (*p* = 0.017), and the mean residual stone volume of the FV-UAS was lower compared to traditional UAS [10].

Zhu et al. performed an experimental study on 9 pigs divided into 3 groups based on different renal perfusion flow rates: 50, 100 and 150 mL/min. They designed a new device for controlling pressure in flexible ureteroscopy (fURS) that incorporates an irrigation/suction platform with pressure feedback control and an access sheath capable of measuring intrarenal pressure (IRP). The researchers found that different flow rates temporarily increased IRP relative to the upper calyceal pressure, but suctioning effectively resolved these increases. The platform renal pelvic outlet pressure readings were relatively stable under different perfusion flow rates for all three groups. These findings suggest that suctioning may be a useful technique for mitigating the negative effects of elevated RPP in clinical settings [11].

Clinical Studies [12,13,14,15,16,17,18,19]. All clinical studies results are summarized in Table 2.

Patent irrigation and platform pressure and UAS [13,14,15,17].

#### 3.1.1. Operative Time and RP

Regarding RIRS, in the studies of Huang et al., Du et al. and Gao et al., [14,15,17] the operative time (OT) ranged from 25 to 80 min, and they all selected the same parameters in the platform: irrigation flow (IF) (PF: 50–150 mL/min), control pressure value was set at −15 to −5 mm Hg, the warning pressure value was set at 20 mm Hg, and the maximum limit value was set at 30 mm Hg. The median OT for Gao et al. was longer (75 min). Factors related to a longer surgical time in the study were multiple stones (54.19%), with 75.81% stones < 30 mm, 66.13% of complex composition and Guy Stone Score (GSS) II 33.87% [17] (see Table 2). For Deng et al., the OT was 24.8 ± 15.9 min, the perfusion flow was set at 100 mL/min, and the RPP control value was set at −2 mmHg. During the procedure, the IRP was controlled below 20 mm Hg, resulting in a clear operative visualization. Additionally, stone particles were effectively removed without the need for stone basketing [13].

#### 3.1.2. Stone Free Rate

Deng et al. reported the immediate and 1st month SFR (90.0–95.6%), and 4% of patients had residual stone >4 mm, requiring subsequent SWL or other management (second ureteroscopy or medication) [13]. Similar results were reported by Huang et al. The immediate SFR was 87.5%, and the 1st month SFR was 97.4% [14]. Gao et al. found that for the patients with a GSS I, the immediate SFR was higher (87%) and the immediate SFR for GSS ≥ II was less than 80% [17]. Du et al. was the only study that reported at 100% SFR in the 1st month for ureteral stones using their modified UAS vs. 87% SFR in the control group [15].

#### 3.1.3. Complications

All studies scored the complications according to the Clavien–Dindo classification [20]. Du et al. reported the lowest percentage of complications in the suction group; only 1.6% postoperative fever was found. None of the cases had a ureteral stricture during 6-month follow-up, and none of the patients required a secondary surgery, contrary to the control group, where 2 cases had intraoperative ureteral perforation, 3 cases had ureteral stricture and 7 cases had postoperative fever [15]. On the other hand, Deng et al. found 14% of Clavien I complications, including pain, fever, nausea/vomiting and tachycardia, and 2% Clavien II; no Clavien III–V complications were noted [13]. Gao et al. reported that the factors associated with major complications were multiple stones (5.95% vs. single: 0%, *p* = 0.002), big stone size (≥50 mm: 13.64% vs. other size: 1.08–5.88%, *p* = 0.028) and high GSS (Grade I vs. VI: 0.59–14.29%, *p* = 0.002) [17].

Suction ureteral access sheath (SUASs) and direct in scope suction (DISS) [12,14,16,18,19].

#### 3.1.4. Operative Time and SFR

In a prospective cohort study, Zeng et al. evaluated the use of a modified access sheath (mUAS) for continuous flow ureteroscopic lithotripsy. The mUAS had a 12/14 Fr diameter and a bifurcated proximal segment, consisting of a straight and an oblique tube at a 45-degree angle. The oblique branch was connected to a negative pressure aspirator with a continuous mode set at 150–200 mm Hg and a flow rate of 60–80 cc/s. The process of stone fragmentation was carried out using the Holmium-YAG laser with preferred settings of 30–35 Hz/0.5–0.6 J. They reported the lowest OT (27.3 min), in contrast to the other 3 studies [16,18,19], with an immediate SFR of 97.3% and 1st month SFR 100% [12].

Zhu et al. used fURS with sUAS created by connecting a channel on the tail of the suctioning UAS to a vacuum device (KYB, China). They showed a longer OT in the sUAS group (49.7 ± 16.3 min) vs. the control group (57.0 ± 14.0 min), *p* < 0.001; however, the immediate SFR in the sUAS was superior (82.4% vs. 71.5%; *p <* 0.02) [16].

Gauhar et al. used a different suction mechanism that was directly connected to the ureteroscope (DISS), which allowed a simultaneous/alternating suction system during/after laser lithotripsy and used the endoscope as a conduit to remove fragments or dust. They mentioned a longer median OT in the DISS group [80 min] vs. the sUAS group (47.5 min), *p* < 0.001; this could be explained because in the DISS group, 40% of patients had multiple stones, and the median stone size was significantly higher at 22 mm (18.0–28.8 mm) vs. 13 mm in the control group (11.8–15 mm), *p* < 0.001. There was no significant difference in the incidence of RF between the DISS group (33.3%) and the other group (35.7% *p* = 0.99); nevertheless, the DISS group (33.3%) required a further RIRS [18].

Qian et al. conducted a prospective cohort with 444 patients with renal stones undergoing fURS, divided in 2 groups, sUAS and no suctioning UAS. They found in the sUAS group a lower OT (72.9 min) vs. in the group no suctioning sUAS (80.0 min), *p* = 0.12. The SFR in the 1st day and 1st month was higher in the sUAS group (86.4–88.9%) vs. the no suctioning sUAS group, which was 71.6–82.7%, *p* = 0.368 [19].

#### 3.1.5. Complications

Zeng et al. found 1.9% fever and 0.9% complication Clavien–Dindo IIIa (false route) [12]. On the other hand, Zhu et al. reported lower total complications in the sUAS group vs. in the traditional group (24.8% vs. 11.5%, respectively; *p* < 0.001) [16]. The most common complications were fever (13.9% vs. 5.5%; *p* = 0.009) and urosepsis (6.7% vs. 1.8%), *p* = 0.029 [16]. Gauhar et al. reported minor complications, not fever, in DISS compared to the control with 17.8% (*p* = 0.05) [18]. Qiang et al. reported the incidence of postoperative fever was lower in the suctioning group (3.7% vs. 14.8%), *p* = 0.030, and SIRS (1.23% vs. 12.3%; *p* = 0.012) [19].

#### 3.1.6. Twitter Results

The use of social media (SoMe) in urology has seen significant growth in recent years. A survey conducted among urological Twitter participants found that 44% agreed that the platform was useful for clinical decision making, and 33% had made a clinical decision after a Twitter discussion. SoMe, particularly Twitter, has become an important tool in academic and research settings [7,21,22]. It allows researchers to share their work with a broader audience, collaborate with peers and promote scientific literacy to the general public.Therefore, researchers should carefully consider the use of different types of media to maximize engagement and ensure responsible use [23].

Social media, has also played a critical role in urology by facilitating physician and patient knowledge acquisition, conference participation and mentorship. Best practices have been established to ensure responsible use, and the COVID-19 pandemic has amplified the role of SoMe in medical education [24]. The European Association of Urology (EAU) Guidelines Office has been a pioneer in using social media to disseminate their recommendations. Through campaigns for awareness days, the EAU Guidelines Office has improved engagement with different audiences, particularly through Twitter, Facebook and Instagram [25]. These findings demonstrate the potential for SoMe to raise awareness, reinforce trust with stakeholders and disseminate scientific information in the medical community [26].

Our Twitter´s search, used the hashtags above mentioned, identified four techniques; there are different techniques of aspiration, but it is mainly about aspiration on the UAS. As the Twitter post by @TFL_URO_APIS [27] suggested, aspiration will be the future, and today we want to know the different systems used.

The first is presented by @DrParimalGharia in pictures and video on his system for keeping a clear view and low IRP during fURS. He connects 2 syringes, one of 10 mL and one of 20 mL, to the water supply circuit, which is activated on demand to aspirate or rinse [28]. This system was also represented in drawings by @AshishRawandale, but only with one syringe [29].

@BEkidneystone presented at the World Congress of Endourology 2019 a poster about the Kalera Vacuum Aspiration Catheter (K-VAC) [30]. This second system K-VAC is a steerable catheter with an outer diameter of 13 Fr and a working length of 72 cm. At the end of the fURS, to evacuate the fragments, the ureteroscope is replaced with a K-VAC device under fluoroscopic guidance to the different calices. It is two coaxial tubes with an active deflective mechanism and also has a built-in on-demand irrigation and aspiration channel; the pressure is between 60 and 80 cmHg, and the suction is controlled with the finger of the surgeon. @PeepeeDoctor presented the first human randomized trial using K-VAC, and in conclusion, the system allows obtaining a higher ablation rate [31]. However, this technique is not done under visual control, but only under fluoroscopic control with an increase in radiation levels.

The Clearpetra Suction-Evacuation is an UAS with a connected suction system. @Abbyqianqxx and @Resistone2022, a medical device and consumables supplier, presented pictures and videos on the technique [32,33,34,35,36], a visible sUAS, in which the sheath diameter can accommodate both the guide wire and the fiber optical ureteroscope, and the procedure can be practiced under the condition of full visibility.

@MeramUrology_ presented at the 7th Istanbul Urolithiasis Days two studies comparing techniques with and without suction [37]. The suction one showed the best renal function in the first days after the operation, with fewer infectious complications and a shorter operative time. The diameter of this system is the same as a standard UAS. @Proximedsrl presented the product with two posts. In a blue test, they put the blue dye on the ureteral sheath and then connected the suction [38], after which the dye was sucked. The other video was on dusting renal stones, the technique most represented on Twitter, which appeared with excellent suction and reduced retropulsion.

In January 2020, @chuikalun1 built a self-made suction device by taking a ureteral access sheath, making a hole on the outer part and connecting a suction hose to it. Two years later, in July 2022, he shared a video of a flexible suction assess sheath: he put it on the ureteroscope, under vision control, using a ureteral access sheath and aspirated dust or used the venture effect to remove the larger fragment. The suction pressure applied to this system is not known [39].

@BioradMedisys congratulated @DocGauhar for winning the 5th Best Affordable New Technologies in the Urology Contest: BANTUC22 for Direct Scope Suction Technique [DISS] [40]. It consisted of putting an adaptor on the irrigation circuit connected to an aspiration in order to use the ureteroscope conduct to remove dust and fragments.

## 4. Discussion

The European Association of Urology recognizes fURS as an alternative for removing stones less than 2 cm in size and as an alternative approach for stones larger than 2 cm. The use of a UAS has been recommended to facilitate fURS and reduce intrarenal pressure [41]. However, smaller-diameter UAS may increase the risk of SIRS, while larger-diameter UAS may result in ureteral trauma and ischemic injury [41,42]. In cases where the intrarenal pressure is significantly high, above 30 mm Hg [43], severe complications, such as urosepsis, shock, and even death, may occur [44].

The incidence of fever during ureteroscopy (URS) has been reported to vary depending on the technique used. In a study by Zeng et al., who used a modified UAS connected to a negative pressure aspirator during URS, only 1.9% of patients developed fever [12], which is consistent with findings from the CROES study [44]. However, this rate is lower than the postoperative fever rates observed by Southern et al. at 6.9% [45]. In contrast, Li et al. reported a much higher incidence of fever (17.5%) after RIRS, with 6.5% of patients developing SIRS [46]. Interestingly, the use of suctioning during URS has been shown to reduce the incidence of fever, even in patients with larger stones (20–30 mm), with rates ranging from 4.3% to 13.1%, and none of these patients developed sepsis, according to studies cited by the authors [13,14].

On the other hand, the stone basketing technique commonly used in traditional fURS can be time-consuming and may leave stones behind [47], leading to the potential formation of a stone street. To mitigate these risks, UAS with suctioning channels has been developed to provide a smaller diameter, improved stone-free rates and improved surgical visualization.

The results of our literature review indicate that various techniques and devices have been used to increase the efficacy of ureteroscopy in the removal of kidney stones. The studies we reviewed included in vitro, preclinical and clinical studies, providing a comprehensive understanding of the current state of the field. In vitro and ex vivo studies (Table 1) revealed that the use of suction during flexible ureteroscopy was effective in removing stone fragments. For example, the combination of a Thulium laser, ureteral access sheath and aspiration resulted in 94% of stone fragments being cleared, with the remaining 6% being less than 2 mm in size [9]. The use of a flexible vacuum-assisted ureteral access sheath was also found to result in higher stone volume clearance rates compared to the traditional ureteral access sheath [10]. In addition, Zhu et al. found that suctioning may be a useful technique for mitigating the negative effects of elevated renal pelvic pressure in clinical settings [11].

Another advantage of the use of sUAS is a significantly higher SFR on the first day postoperatively. According to a study by the Endourology Disease Group for Excellence (EDGE consortium), fragment sizes larger than 4 mm after URS are linked to higher rates of stone growth, complications and the need for additional surgical procedures. As a result, the EDGE consortium recommends aiming for complete stone-free status during stone surgery, regardless of whether the stones are extracted using the dusting or basketing method [48].

The clinical studies reviewed confirm the advantages of aspiration during ureteroscopy. Deng et al. found that the intrarenal pressure was controlled below 20 mm Hg during the procedure, leading to clear operative visualization and effective removal of stone particles without the need for stone basketing [13]. Huang et al., Du et al. and Gao et al. all found that using platform pressure and UAS during ureteroscopy improved the SFR [14,15,17].

Different suction modalities during URS have been evaluated in experimental and clinical studies and appear to offer several benefits, including improved SFR and intraoperative visibility and decreased OT and complications. However, clinical evidence supporting the routine use of suction during URS is still weak. Moreover, there is no consensus regarding the best suction technology in clinical practice as far as the different suction modalities that have been developed and proposed. Further multicentric and prospective studies, with an adequate number of patients and standardized outcomes assessments, are needed to confirm these results.

Certain limitations of the current study must be acknowledged. First, the evidence of the review is limited by the small sample sizes of the studies and the lack of long-term follow-up data. Second, the studies included in the review were conducted in various settings, with varying patient populations and using different techniques, making it difficult to generalize the results to a broader population.

The results of the present narrative review suggest that the use of suction in flexible ureteroscopy may be an effective technique for removing stone fragments. However, further research is needed to confirm these results and to determine the optimal use of suction in flexible ureteroscopy, including the ideal pressure settings, flow rates and access sheath size. Additionally, long-term follow-up data are needed to assess the safety and effectiveness of the technique, including the incidence of adverse events and complications. The results of this review highlight the need for high-quality, well-designed clinical studies to inform the development of clinical practice guidelines and policies related to the use of suction in flexible ureteroscopy.

## 5. Conclusions

In conclusion, the use of suction during flexible ureteroscopy has shown promising results in improving success rates and reducing complications in the removal of kidney stones. The studies reviewed indicate that suctioning can increase stone-free rates, control intrarenal pressure and improve surgical visualization. However, more clinical evidence is needed to determine the optimal use of suction in fURS and to assess its long-term safety and effectiveness. Further high-quality, well-designed clinical studies are necessary to inform clinical practice guidelines and policies related to the use of suction in fURS.

## Figures and Tables

**Table 1:** In vitro studies included.

Part A					
**Authors**	**Accrual Ys**	**Center**	**Study Type**	**Sample**	**Groups of** **Comparison**
Schneider et al. [8]	2020	United States	In vitro	Phantom stones A: 0.5 and <1 mm B: <0.5 mm. Both <1 and <0.5 mm groups	-
Jiang et al. [9]	2021	United States	Ex vivo	72 ex vivo porcine kidney ureter models	Holmiun vs. MOSES vs. TFLSuction vs. no suction UAS 14 fr vs. no UAS
Chen et al. [10]	2022	China	Experimental	20 porcine kidney	1–10: FV-UAS group 11–20: traditional UAS group
Zhu et al. [11]	2016	China	Experimental	9 pigs 18 kidney	sUAS: with pressure/ suctioning/working channel
**Part B**	**Continue**	**In Vitro Studies**			
**Author**	**Suction Modality**	**Operative Time**	**Stone Free Rate**	**Definition of Stone Free Rate**	
Schneider et al. [8]	Standard Luer Lock syringe through the working channel of a fURS	Mean time: <0.5 mm: 2 min 52 s <1 mm: 8 min 53 s *p* = 0.003	% Stone fragments Suction <0.5 mm: 86% = <1 mm 86% Fragments trapped in wc: <0.5 vs. <1 mm= 64% vs. 78% *p* = 0.001	Phantom stones A: 0.5 and <1 mm B: <0.5 mm. Both <1 and <0.5 mm groups	
Jiang et al. [9]	Dual lumen ureteroscope with suction port 3.3 Fr	URS 8.6 ± 4.3 min	94.3% (sTFL + suction + UAS) vs. 64.8% (Ho:YAG + no suction + no UAS): *p* ≤ 0.01 Aspiration without UAS improve stone clearance 17.3% TFL *p* ≤ 0.02	Fragments <100 micras (i.e., dust).	
Chen et al. [10]	Flexible vacuum assistant UAS (FV-UAS) 12/14F, 46 cm	FV-UAS vs. traditional UAS group: 44.2 vs. 39.7 *p* = 0.1	Residual stone vol: FV-UAS vs. traditional UAS group: 33.7 vs. 92.5 *p* = 0.017 Stone clearance FV-UAS vs. traditional UAS groups: 98.5% vs. 95.9% *p* = 0.017 Complete SFR: 70% FV-UAS	Stone volume clearance rate: 1-(residual stone vol/preop stone vol) × 100%	
Zhu et al. [11]	-	-	RPP were similar to those recorded by the invasive blood pressure monitor. RPP from the renal pelvic outlet were similar to those from the upper calyceal area	-	

**Table 2:** Clinical studies included.

Part A					
Authors	Accrual Ys	Center	Study Type	Sample	Groups of Comparison
Zeng et al. [12]	2016	China	Prospective	104: Upper: 52 Middle: 19 Lower: 32 Size of the stones was 73.91 ± 66.25 mm^2^	-
Deng et al. [13]	2016	China	Retrospective	93: 69 renal 17 ureteral calculi 7 both	
Huang et al. [14]	2017	China	Prospective	40 patients with a solitary kidney	-
Du et al. [15]	2018	China	Prospective	122 patients with large ureteral stones below L4 level	62 study group vs. 60: control group
Zhu et al. [16]	2018	China	Prospective	165	URS + sUAS
Gao et al. [17]	2022	China	Retrospective	278	Suctioning fURS + Intelligent pressure-control
Gauhar et al. [18]	2022	Singapore	Prospective	58: 30 DISS28 SUAS	Direct in-scope suction (DISS) technique
Qian et al. [19]	2022	China	Prospective (2017–2019)	444	sUAS
**Part B**	**Continue**	**Clinical**	**studies**		
**Authors**	**Suction modality**	**Operative time**	**Stone free rate**	**Definition of Stone free rate**	**Complications**
Zeng et al. [12]	mUAS 12/14 Fr Negative pressure aspirator set to continuous mode at 150–200 mmHg.	27.3 min	Immediate SFR:97.3%. 1st month SFR: 100%.	No stone fragment on the fluoroscopy/KUB.	Successful mUAS insertion: 77.1% False route: 0.9% (Clavien IIIa) Fever: 1.9% (Clavien I)
Deng et al. [13]	Patented irrigation + suctioning platform + UAS	24.8 ± 15.9 min	Mean stone size: 15.9–5.2 mm Immediate SFR: 90.0% 1st month: 95.6%.	-	Fever 4.3% Clavien I: 14.4 % Clavien II: 2% Clavien III-IV: 0%
Huang et al. [14]	Patented irrigation + suctioning platform + UAS	25.2 ± 14.5 min	Stone sizes ranged from 0.9 to 3.0 cm. SFR 1 month: 87.5% SFR 3 month: 92.5%	Stone fragments < 4 mm	Fever: 5%
Du et al. [15]	The study group: sUAS 12/14 Fr 30–45 cm + pressure monitor vs. The control group: URS + without suction	25.3 vs. 47.2 *p* = 0.00	SFR 1 month Study group: 100% vs. control group: 81.7% *p* = 0.00	Stone fragments < 4 mm	Study group vs. control group Fever: 1.6% vs. 11%Ureteral stricture: 0 vs. 3% *p* = 0.08 Secondary surgery: 0 vs. 7% *p* = 0.01
Zhu et al. [16]	URS without sUAS	Study vs. traditional group 49.7 ± 16.3 min vs. 57.0 ± 14.0 min *p* = < 0.001	Study group vs. traditional group: 1st day postoperatively 82.4% vs. 71.5% *p* = 0.02.	Stone fragments < 2 mm	Study vs. traditional group: overall complications 11.5% vs. 24.8% *p* = 0.001 Fever: 5.5% vs. 13.9% *p* = 0.009) Urosepsis requiring only additional antibiotics: 1.8% vs. 6.7%; *p* = 0.029).
Gao et al. [17]	2022	-	75 min.	SFR immediate: 80.65% SFR 1 month: 82.26% Patients with a stone size <40 mm and a Guy's SS GI: SFR immediate 87%	Stone fragments < 4 mm X-ray
Gauhar et al. [18]	2022	Control: sUAS 11/13 Fr	>DISS group 80 min vs. SUAS group 47.5 min (*p* < 0.001).	RF: DISS vs. sUAS: 33.3% vs. 35.7% *p* = 0.99 33.3% DISS: required a further RIRS	3 months KUB/US/Scan:absence of a single residual fragment >3 mm or in the absence of multiple fragments of any size.
Qian et al. [19]	2022	Without sUAS	Group sUAS vs. no sUAS: 72.9 (28.1) min vs. 80.0 (29.5) min; *p* = 0.121	Group sUAS vs. no sUAS: 1st day SFR: 86.4% vs. 71.6%; *p* = 0.034 1st month 88.9% vs. 82.7%; *p* = 0.368.	Stone fragments < 4 mm X-ray
